# Three Innovative Green and High-Throughput Microwell Spectrophotometric Methods for the Quantitation of Ceritinib, a Potent Drug for the Treatment of ALK-Positive Non-Small Cell Lung Cancer: An Application to the Analysis of Capsules and Drug Uniformity Testing

**DOI:** 10.3390/molecules28207054

**Published:** 2023-10-12

**Authors:** Reem M. Abuhejail, Nourah Z. Alzoman, Ibrahim A. Darwish

**Affiliations:** Department of Pharmaceutical Chemistry, College of Pharmacy, King Saud University, P.O. Box 2457, Riyadh 11451, Saudi Arabia

**Keywords:** ceritinib, microwell analysis, spectrophotometry, green analytical approach, high-throughput analysis

## Abstract

Ceritinib (CER) is a potent drug that has been recently approved by the Food and Drug Administration for the treatment of patients with non-small cell lung cancer harboring the anaplastic lymphoma kinase mutation gene. The existing methods for the quality control of CER are very limited and suffer from limited analytical throughput and do not meet the requirements of the green analytical principles. This study presented the first-ever development and validation of three innovative green and high-throughput microwell spectrophotometric methods (MW-SPMs) for the quality control of CER in its dosage form (Zykadia^®^ capsules). These MW-SPMs were based on the formation of colored *N*-vinylamino-substituted haloquinone derivatives of CER upon its reactions with each of chloranil, bromanil, and 2,3-dichloro-1,4-naphthoquinone in the presence of acetaldehyde. The optimized procedures of the MW-SPMs were established, and their analytical performances were validated according to the ICH. The linear range of the MW-SPMs was 5–150 µg/mL, with limits of quantitation of 5.3–7.6 µg/mL. The accuracy and precision of the MW-SPMs were proved, as the average recovery values were 99.9–101.0%, and the relative standard deviations did not exceed 1.8%. The three methods were applied to the determination of CER content in Zykadia^®^ capsules and drug content uniformity testing. The greenness of the MW-SPMs was proved using three different metric tools. In addition, these methods encompassed the advantage of high-throughput analysis. In conclusion, the three methods are valuable tools for convenient and reliable application in the pharmaceutical quality control units for CER-containing capsules.

## 1. Introduction

Non-small cell lung cancer (NSCLC) is the most prevalent form of lung cancer, accounting for approximately 85% of all cases. It is a malignant disease primarily affecting the cells that line the lung airways. It involves the uncontrolled growth and spread of abnormal cells, resulting in tumor formation [1]. The management of NSCLC depends on several factors, such as cancer stage, overall health of the patient, and specific tumor characteristics. In cases of advanced metastatic or localized NSCLC, the initial treatment option is chemotherapy using first-generation tyrosine kinase inhibitors (TKIs), like gefitinib and erlotinib [2,3,4]. However, a considerable number of NSCLC cases exhibit a chromosomal rearrangement that fuses the EML4 (echinoderm microtubule-associated protein-like 4) gene with the ALK (anaplastic lymphoma kinase) gene. The resulting fusion gene leads to the reactivation of the kinase enzyme, contributing to increased cancer development and driving the malignant features of the cancer cells. Unfortunately, the first-generation TKIs are not effective in inhibiting the kinase activity of the EML4-ALK fusion protein [4,5]. Based on this finding, pharmaceutical companies have undertaken research programs to discover and develop effective ALK inhibitors.

**Figure 1:**
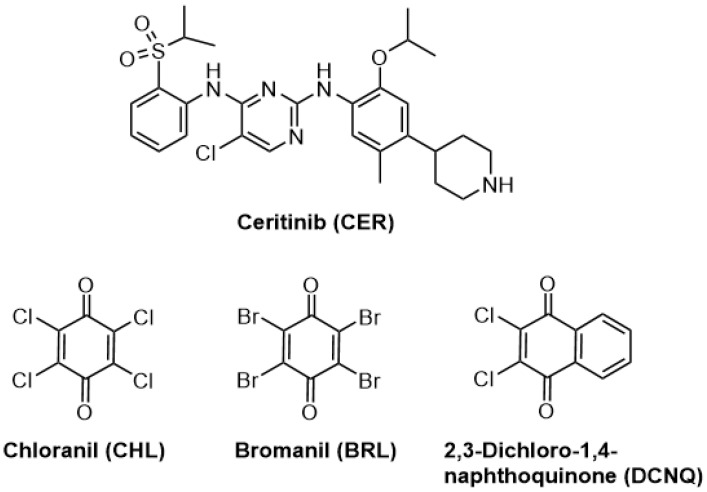
The chemical structures of ceritinib (CER), chloranil (CHL), bromanil (BRL), and 2,3-dichlor-1,4-onaphthoquinone (DCNQ).

During the process of discovering new drugs, Novartis Pharmaceutical Corporation (located in Basel, Switzerland) identified ceritinib (CER) as a potent inhibitor of ALK. The chemical structure of CER is displayed in Figure 1 [6]. CER was specifically designed to overcome resistance to the earlier ALK inhibitor, crizotinib. It has received approval from the U.S. Food and Drug Administration (FDA) as a breakthrough treatment option for patients with ALK-positive NSCLC. CER is marketed under the trade name Zykadia^®^ capsules [7]. Since its approval, CER has demonstrated remarkable effectiveness and safety, providing renewed hope for patients with this particular subtype of lung cancer. CER has the ability to inhibit multiple receptor tyrosine kinases, including ALK, ROS1, and insulin-like growth factor 1 receptor (IGF-1R). By specifically targeting and blocking the activity of these kinases, CER effectively suppresses the growth and survival of cancer cells that carry ALK gene rearrangements. This targeted approach makes CER an extraordinarily potent and highly specific therapy for ALK-positive NSCLC [8].

While CER has shown significant clinical benefits, it is also associated with certain adverse events, including gastrointestinal disturbances, hepatotoxicity, fatigue, and edema. These side effects can be managed by adjusting the dosage; however, CER has also been linked to QT interval prolongation, which requires careful monitoring and critical dose management [8]. Therefore, ensuring the precise control of CER content in its dosage form (Zykadia^®^ capsules) is crucial to achieve a therapy that is both effective and safe with CER. To accomplish this objective, the development of an efficient and reliable analytical tool is necessary for accurately quantifying the amount of CER present in the capsules.

There are limited analytical methods available for characterizing and quantifying CER in bulk drug or capsules. These methods include liquid chromatography coupled with an ultraviolet detector (HPLC-UV) [9,10] or a tandem mass spectrometric detector (LC-MS/MS) [11], and voltammetry [12]. HPLC-UV methods were primarily developed for stability testing of CER, whereas LC-MS/MS methods were designed for quantifying genotoxic impurities in CER bulk form [11]. Although liquid chromatographic methods offer versatility in specific scenarios, they come with several disadvantages and limitations when applied in pharmaceutical quality control laboratories for characterizing CER formulation. The drawbacks of liquid chromatographic methods used in pharmaceutical quality control laboratories for drug formulation characterization include the following: (1) Complexity: The technique requires a high level of expertise to operate the instrument, optimize the method, and accurately interpret the results. (2) High cost: There are significant expenses involved in setting up and maintaining the system, as well as acquiring consumables. (3) Challenges in high-throughput situations: Liquid chromatography methods can be time-consuming, posing difficulties in rapidly processing numerous samples, such as in dosage form uniformity testing. (4) Solvent disposal challenges: The use of organic solvents in liquid chromatography methods presents issues related to hazardous waste disposal, which can be environmentally unfriendly. To overcome these disadvantages, it is crucial to develop alternative methodologies that can provide better solutions.

Microwell spectrophotometric methods (MW-SPMs), assisted with absorbance microplate readers, have gained significant importance in pharmaceutical analysis due to their exceptional advantages [13,14,15]. One key benefit underlying the MW-SPMs is their ability to utilize smaller sample volumes compared to conventional spectrophotometric techniques, which rely on volumetric flasks and cuvettes. This approach reduces waste generation during the analytical process and lowers the cost of reagents. Additionally, it minimizes the use of hazardous solvents and chemicals, promoting an eco-friendly and sustainable approach. The versatility of the MW-SPMs allows for a wide range of applications in various pharmaceutical industry activities. These methods can be easily automated using robotic systems, enhancing their efficiency, reducing errors, and saving time and labor in the laboratory. By facilitating the rapid analysis of multiple samples, these methods contribute to achieving uniformity in pharmaceutical formulations and other pharmaceutical industry activities. These advantages have led to the increasing popularity of employing MW-SPMs not only in the pharmaceutical industry but also in other fields.

This study has presented the first-ever development of three distinct MW-SPMs for the quality control of CER in Zykadia^®^ capsules. In these methods, colored N-vinylamino-substituted haloquinone derivatives of CER were formed within the microwells through reactions of CER with haloquinone reagents in the presence of acetaldehyde (ACD). These haloquinone reagents were chloranil (CHL), bromanil (BRL), and 2,3-dichloro-1,4-naphthoquinone reagents.

## 2. Results and Discussion

### 2.1. Strategy for Selection of Color-Forming Reactions

**Figure 2:**
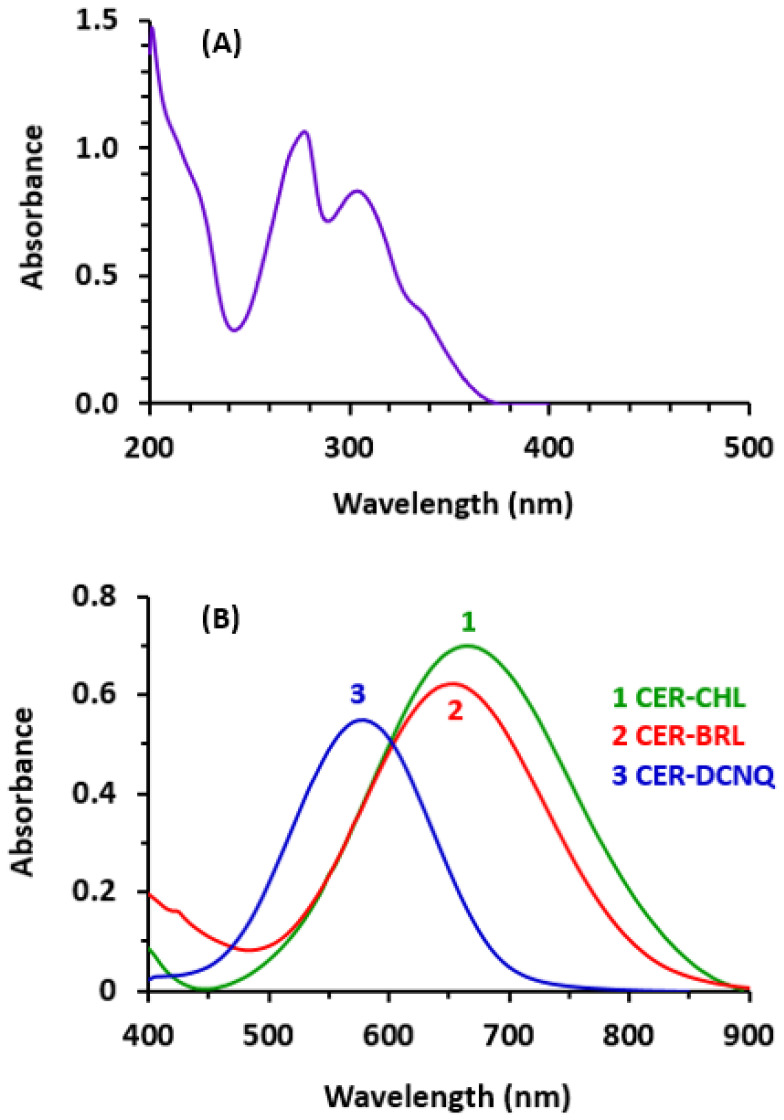
The absorption spectra of CER (**A**) and its reaction products with CHL, BRL, and DCNQ, in the presence of ACD (**B**). The concentration of CER solution alone (without reaction) was 25 µg/mL. The concentration of CER in its reaction’s mixtures was 25 µg/mL. All solutions were in methanol.

The UV absorption spectrum of CER solution (Figure 2A) exhibited a forked maximum absorption peak (λ_max_) at 277 nm and 304 nm, and an absorption cut-off at 370 nm. According to these spectral characteristics, it might be possible to determine CER by measuring its UV absorption. However, the development of a method based on measuring a visible light of colored derivative of CER would provide more advantages over UV measurements [16]. These advantages include the following: (1) a broader wavelength range, enabling the employment of a wider range of color-forming derivatization reactions, (2) adaptability to the more encountered automated colorimeters and absorbance microplate readers for high-throughput analysis, where rapid results are required under industrial settings or quality control laboratories, and (3) being less susceptible to interference from pharmaceutical inactive ingredients or any turbidity in solutions. To achieve these advantages for the analysis of CER, it should be transformed into colored derivatives via the color-producing reaction. 

**Figure 3:**
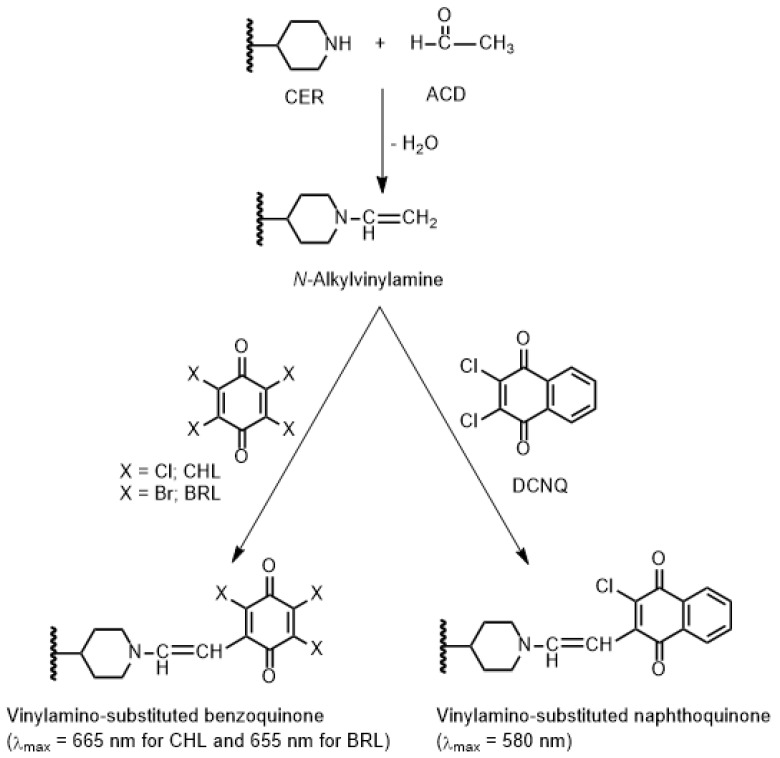
Scheme for the reaction pathway of CER with ACD and each of CHL, BRL, and DCNQ.

The chemical structure of CER (Figure 1) contains a piperidinyl amino group. This amino group could be reacted with many reagents, producing colored derivatives [17,18,19,20]. Among these reagents, haloquinone reagents are widely used as chromogenic reagents via different reaction mechanisms. The most versatile reaction of haloquinone reagents is their reactions with secondary amines, in the presence of ACD, producing colored *N*-vinylamino-substituted haloquinone derivatives. The key advantage of this reaction is its rapid occurrence under very mild conditions, and accordingly, it might be employed for high-throughput analysis. This reaction has not been described so far for CER; therefore, it was considered in this study as a color reaction for CER. This reaction was based on the interaction between the piperidinyl amino group of CER with ACD, forming a *N*-alkylvinylamine derivative of CER, which subsequently reacted with CHL, BRL, and DCNQ to produce colored vinylamino-substituted haloquinone derivatives. The reaction scheme is illustrated in Figure 3.

### 2.2. Methodology Selection and Design

The MW-SPMs described in this study utilized a 96-microwell-based analysis interfaced with an absorbance microplate reader. This methodology has become highly valuable in pharmaceutical analysis for its alignment with green chemistry approaches [21,22,23], and for its capability for high-throughput analysis [24,25]. This methodology offers numerous advantages, including miniaturization, automation, parallelization, and significant advancements in both the environmental sustainability and efficient analysis of large sample sets [26,27,28]. Microwell analysis contributes to green chemistry in several ways: (1) it reduces sample and reagent consumption, leading to less waste generation and a lower environmental impact; (2) it optimizes energy utilization by enabling faster and more efficient analysis due to the simultaneous nature of miniaturized analysis; and (3) microwell assays are compatible with automation, ensuring high precision and reproducibility of analytical results by minimizing human errors.

In the context of high-throughput analysis, microwell methods offer several benefits. These include the following: (1) simultaneous analysis facilitating the parallelization of measurements, allowing for the analysis of multiple samples, (2) miniaturized format: microwell methods utilize small sample and reagent volumes due to their miniaturized format, which enables the analysis of a vast number of samples even with limited resources, making it a cost-effective approach, and (3) ease of data handling and analysis, as the microwell analysis generates large datasets, and they can be efficiently handled and analyzed using automated data analysis software. Given these advantages, the present study focused on developing the MW-SPMs for the quantitation of CER in its capsules. 

The proposed MW-SPMs involved conducting the aforementioned reactions of CER in transparent 96-well plates, and their absorbances were simultaneously measured using an absorbance microplate reader.

### 2.3. Absorption Spectra and Reaction Stoichiometry 

Upon mixing CER with ACD, followed by CHL, BRL, and DCNQ, the reaction mixtures turned red (with CHL and BRL) and yellow-orange (with DCNQ). The absorption spectra of the reaction mixtures were recorded against reagent blanks. The spectra showed maximum absorption peaks (λ_max_) at 665 nm, 655 nm, and 580 nm for the reaction mixtures involving CHL, BRL, and DCNQ, respectively (Figure 2B). Obviously, the λ_max_ of the reaction products were significantly red shifted from that of the underivatized CER. This shift enabled the measurements to be conducted in the visible region and eliminates the limitations arising from measuring in the UV region. It was also observed that the absorption intensities of these new bands increased with increasing concentrations of CER. The emergence of these new bands, and their dependence on the concentrations of CER, provided evidence of reaction occurrence. The shapes and patterns of these new absorption bands closely resembled those reported in the literature for the condensation reaction products of other amines with CHL, BRL, and DCNQ [20]. Additionally, acidification of the reaction mixtures with dilute mineral acid (HCl) did not cause fading of the solutions’ colors. This observation revealed that the reaction proceeded via irreversible covalent bonding, rather than the possible irreversible charge-transfer reaction between CER, as an electron donor, and the reagents (CHL, BRL, and DCNQ), as electron acceptors [29]. 

### 2.4. Optimization of Reaction Conditions

**Figure 4:**
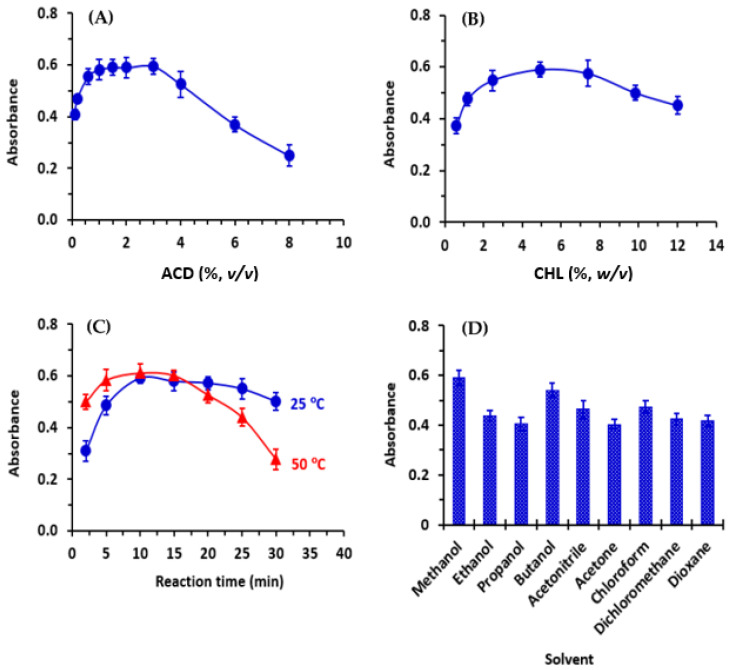
The effect of ACD concentration (**A**), CHL concentration (**B**), reaction times at 25 °C and 50 °C (**C**), and solvent (**D**) on the absorbances of reactions of CER with CHL in the presence of ACD. The concentration of CER in the reactions was 30 µg/mL. The reactions were carried out in methanol.

Sets of experiments were conducted to investigate and optimize the factors affecting the reactions described in this study. These factors included the concentrations of ACD and haloquinone reagent (CHL, BRL, and DCNQ), reaction time, temperature, and solvent. Each variable was altered individually, while keeping the others constant. Figure 4 illustrates the results obtained using the CHL reagent, while similar results were obtained for BRL and DCNQ.

The study involving the effect of reagents revealed that the color development depended on both ACD and haloquinone reagents (Figure 4A,B, respectively). The highest absorbances were achieved when the concentration of ACD was in the range of 1–3% (*v*/*v*). A concentration of 2% (*v*/*v*) was chosen for further experiments. Similarly, the optimal concentration of haloquinone reagent was found to be in the range of 3–7% (*w*/*v*). Thus, a concentration of 5% (*w*/*v*) was selected for further investigations. 

To determine the appropriate reaction time, reactions were allowed to proceed for varying time periods at room temperature (25 ± 2 °C) and at 50 °C. Higher temperatures were not investigated, as the maximum controllable temperature using the temperature controller of a microplate reader was 50 °C were conducted at different durations. The results indicated that complete color development occurred after 5 and 10 min at room temperature and 50 °C, respectively (Figure 4C). Prolonging the reaction time had no effect on color development at room temperature, but it negatively impacted the reaction at 50 °C. Consequently, further experiments were carried out at room temperature for 10 min. 

In order to select the most suitable solvent, several solvents were assessed (Figure 4D). The highest color intensity was obtained when methanol was used; hence, it was chosen for subsequent investigations.

### 2.5. Development and Validation of the MW-SPMs

Table 1 summarizes the optimal conditions for conducting the aforementioned reactions of CER in a 96-microwell assay plate. The absorbances were measured using an absorbance microplate reader at 665 nm, 655 nm, and 580 nm for reactions with CHL, BRL, and DCNQ, respectively. The proposed MW-SPMs, for the quantitative determination of CER, were validated according to ICH guidelines for the validation of analytical procedures [30].

#### 2.5.1. Linear Range and Sensitivity

**Figure 5:**
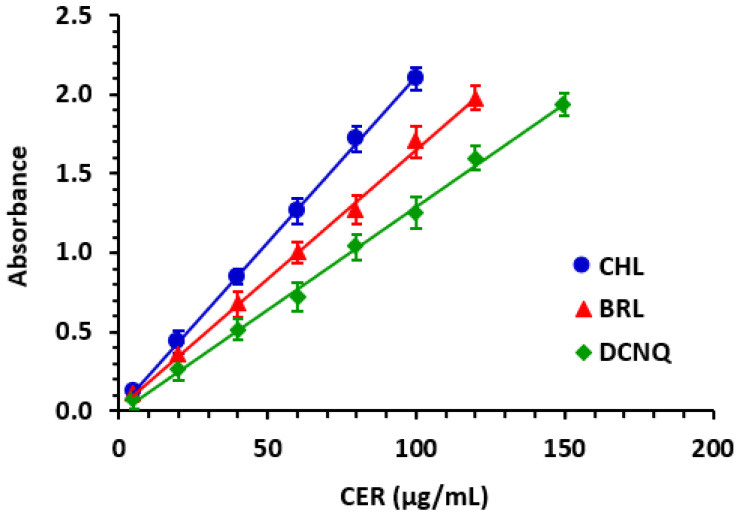
The calibration curves for the quantitation of CER using the proposed MW-SPMs via its reactions with ACD and each of CHL, BRL, and DCNQ.

The calibration curves relating the absorbances of the CER reaction solutions with their corresponding CER concentrations were generated for the three methods involving CHL, BRL, and DCNQ reagents (Figure 5). The data were subjected to linear fitting, and linear equations were derived, along with their corresponding parameters (intercepts, slopes, and determination coefficients). The obtained values are presented in Table 2. For the quantitation of CER, the proposed MW-SPMs demonstrated linearity within the ranges of 5–100 µg/mL, 5–120 µg/mL, and 5–150 µg/mL for the methods using CHL, BRL, and DCNQ, respectively. The linearity of these methods was excellent, as the determination coefficient (r^2^) values were ≥0.9991.

To assess the sensitivity of the methods, the limit of detection (LOD) and limit of quantitation (LOQ) were determined, following the procedures outlined in the ICH guidelines for the validation of analytical procedures [30]. The LOD values were found to be 2.2 µg/well and 3.4 µg/well for the methods employing the OBQ and DDQ reactions, respectively. Furthermore, the LOQ values were determined to be 5.3 µg/mL, 5.9 µg/mL, and 7.6 µg/mL for the methods involving the reactions with CHL, BRL, and DCNQ, respectively.

#### 2.5.2. Accuracy and Precision

The intra- and inter-day accuracy and precision of the MW-SPMs were assessed by analyzing replicate sample solutions of CER solutions containing different concentrations (low, medium, and high). The results obtained, which were expressed as recovery percentages and their relative standard deviations (RSDs), were used as measures for accuracy and precision, respectively.

In the case of intra-day analysis, the replicate samples (n = 3) were simultaneously analyzed as a batch within a single day on one assay plate. For inter-day analysis, duplicate samples of each concentration level were analyzed over the course of three consecutive days. The averages of recovery percentages and their RSD were calculated, and the values are given in Table 3. The average recovery percentages were ≥99.9%, and the RSD values did not exceed 1.8%. The high recovery (%) and low RSD values revealed the high accuracy and precision, respectively.

The high accuracy and precision of the proposed MW-SPMs could be attributed to four key factors. These factors are: (1) A controlled environment, as the methods were performed in a controlled environment, which promote stable reaction conditions and absorbance measurements throughout the analysis. (2) Utilization of microwell plates allows for the precise manipulation and confinement of samples, reducing their variability, and enabling accurate measurements. (3) The use of very small sample volumes in the MW-SPMs helps reduce the potential for errors, decreasing the likelihood of inaccuracies introduced during sample preparation or transfer. (4) Prevention of cross-contamination, as the segregation of samples into individual wells in the MW-SPMs prevents cross-contamination between the samples. This isolation ensures that measurements or reactions solely take place within the designated wells, eliminating the risk of sample mixing. Consequently, the accuracy of the methods is enhanced. Taken together, these factors contributed towards the high precision and accuracy observed in the proposed MW-SPMs, making them reliable tools for the quantitation of CER.

#### 2.5.3. Specificity

The proposed MW-SPMs demonstrated a high level of specificity for the quantitation of CER in Zykadia^®^ capsules, effectively eliminating interference from the inactive ingredients present in the capsule’s shell. This specificity was attributed to two main factors. Firstly, the measurements were conducted in the visible region of the electromagnetic light spectrum. These wavelengths are far from the UV-absorbing properties of the inactive ingredients in the capsules. Secondly, the use of methanol for the dissolution and preparation of the capsule sample solution played a crucial role in achieving specificity. Methanol was selected, as it selectively dissolves CER, while leaving the inactive ingredients undissolved. This is because the inactive ingredients typically dissolve in water, but do not dissolve in methanol. 

#### 2.5.4. Robustness and Ruggedness

The robustness of the proposed MW-SPMs, which refers to the impact of minor changes in procedure conditions on their analytical performance (precision and accuracy), was evaluated. The changes considered were the concentrations of ACD and haloquinone reagent (CHL, BRL, and DCNQ), along with the reaction time. These conditions were changed in the ranges outlined in Table 4. 

The evaluation revealed that these minor changes did not have a detrimental effect on the performance of these methods. The recovery values ranged from 98.8% to 102.9%, with the RSD values ranging from 0.6% to 1.7%. These results clearly demonstrate the robustness of these methods and their suitability for the routine and comfortable analysis of CER.

The ruggedness of these methods was also assessed in terms of analyst-to-analyst and day-to-day reproducibility. The RSD values observed during this assessment did not exceed 1.8%, providing further evidence that the proposed methods are rugged and capable of consistently delivering accurate and precise results.

In summary, the evaluations of robustness and ruggedness confirm the reliability and suitability of the proposed MW-SPMs for the routine analysis of CER. They exhibit resilience to minor variations in method variables and demonstrate a consistent performance across different analysts and days, ensuring the robustness and reliability of the methods.

### 2.6. Analysis of Zykadia^®^ Capsules and Content Uniformity Testing

After successfully validating the suitability of the proposed MW-SPMs for quantifying CER in Zykadia^®^ capsules, these methods were applied to determine the concentrations of CER in capsule samples at different predetermined levels (Table 5). The results demonstrated label claim percentages of mean values, with 100.1 ± 0.3, 100.3 ± 1.3, and 100.5 ± 0.6 obtained using the CHL, BRL, and DCNQ methods, respectively (Table 5). These high label claim percentages indicate the effectiveness of the MW-SPMs in accurately and precisely determining the CER content in Zykadia^®^ capsules.

To assess content uniformity, the testing followed USP guidelines [31]. Ten capsules were individually analyzed using the proposed three MW-SPMs to determine their CER contents. Acceptance values were calculated and found to be 4.6, 4.9, and 5.6 for the CHL, BRL, and DCNQ methods, respectively (Table 6). These values were below the maximum allowed value of 15, confirming the excellent uniformity of CER contents in the capsules and ensuring drug uniformity.

### 2.7. Greenness Levels of the MW-SPMs

Principally, the microwell methods assisted with microplate readers align with the principles of green analytical chemistry (GAC) by downsizing analytical processes [12,13,15]. This approach offers numerous benefits compared to traditional spectrophotometric methods that employ volumetric flasks and cuvettes. These advantages include the utilization of smaller sample and reagent volumes, as well as a reduction in waste generation. To assess the environmental sustainability and greenness of the proposed MW-SPMs, three effective metric tools were employed: the analytical eco-scale (AES) approach [32], the green analytical procedure index (GAPI) [33], and AGREE—analytical greenness metric approach and software [34]. These tools provide precise and comprehensive evaluations of the ecological impact of analytical procedures, and detailed information regarding their evaluation parameters can be found in the corresponding published articles [32,33,34]. 

The results obtained using the AES approach are shown in Table 7 for the three proposed methods, as they involved the same reagent category, procedures, and operating conditions. Penalty points (PPs) were assigned for the solvent (methanol) and reagents (CHL, BRL, and DCNQ), with one PP for each, resulting in a subtotal of two PPs. The hazardous effects of methanol and reagents contributed to a subtotal of six PPs. Parameters related to energy consumption via the instrument and occupational hazards did not receive any PPs, as they complied with the tool guidelines [32]. Waste production and treatment parameters were assigned subtotals of one and three PPs, respectively. These scores were assigned, as the proposed MW-SPMs generated less than 1 mL of waste per sample, and the waste was not treated. The total PPs for the MW-SPMs amounted to 12, resulting in an eco-scale score of 88 (100 − 12). This high score indicates the excellent level of environmental friendliness of the proposed MW-SPMs, in accordance with the metric tool guidelines [32].

**Figure 6:**
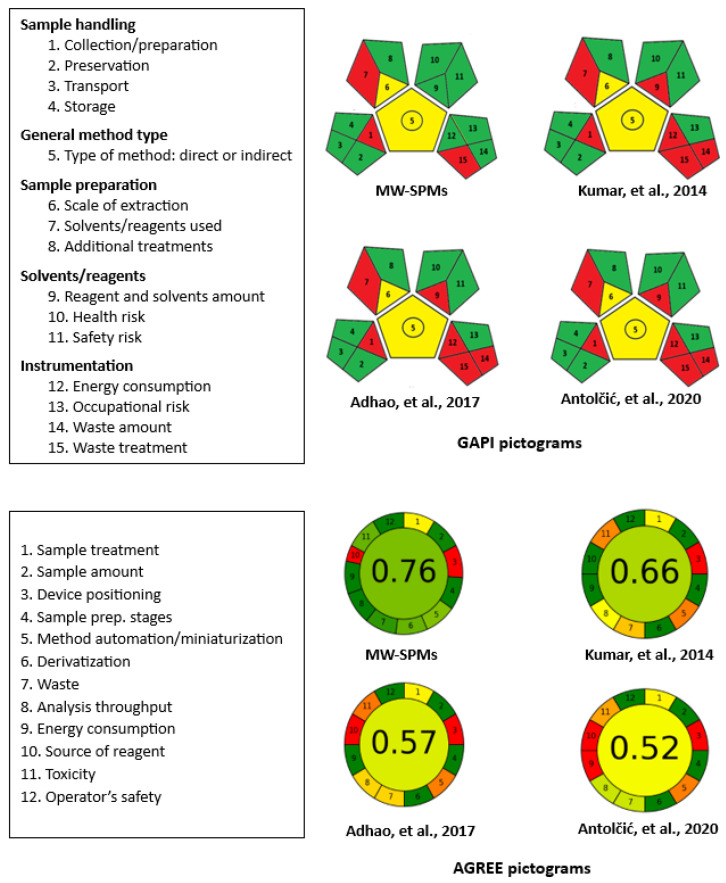
Comparative results of the GAPI and AGREE metric tools for evaluation of the greenness of the proposed MW-SPMs and reported methods (published by the authors of [9,10,11]) for the determination of CER.

The results obtained using the GAPI tool are given in Figure 6 (upper section), represented as a pictogram of fifteen evaluation parameters of six categories. Of these 15 parameters, three (parameters 1, 7, and 15) were colored red in the pictogram. The red color indicates that these parameters did not meet the greenness criteria. Parameter 1 signified that the samples were collected or prepared in an off-line manner. Parameter 7 indicated the use of methanol for sample preparation. Parameter 15 highlighted that the waste generated using the assay was not treated. Parameters 5 and 6 were assigned a yellow color, indicating partial compliance with the green criteria. Parameter 5 signified that the methods are suitable for quantitative analysis, while parameter 6 indicated that the sample extraction was conducted on a microscale. The remaining parameters were colored green, indicating that they fully met the requirements of green procedures, according to the guidelines of the GAPI tool [33].

The pictogram that was generated using the AGREE tool is presented in Figure 6 (lower section). Parameter 1 (related to sample treatment) was represented in yellow, as the samples were treated manually. Parameter 3 (related to device positioning, either on-line or off-line) and parameter 10 (related to the source of reagent) were depicted in red, as the analysis was conducted off-line using a plate reader and with reagents of a chemical source, respectively. The remaining parameters were represented in green. The total score obtained was 0.76 out of 1, indicating a high level of greenness for the proposed MW-SPMs.

In conclusion, these results obtained using these three tools confirm the greenness of the proposed methods and their adherence to the principles of GAC.

### 2.8. Comparison of the Greenness Levels of the MW-SPMs with the Reported Methods

The levels of greenness in the proposed MW-SPMs were compared to those of previously reported liquid chromatographic methods for CER [9,10,11]. This comparison was carried out using the GAPI and AGREE tools, and the obtained pictograms for the reported methods are shown in Figure 6. The GAPI tool revealed similar greenness levels for the reported methods, which were lower than that of the proposed MW-SPMs, particularly for parameters 9, 12, and 14 (corresponding to reagents and solvent amount, instrument energy consumption, and waste amount, respectively). This observation can be attributed to the fact that liquid chromatography consumes larger quantities of solvents and reagents, requires more energy for its operation, and generates larger volumes of waste compared to the proposed MW-SPMs.

The AGREE tool, which is the most recent and comprehensive tool, determined that the proposed MW-SPA exhibited the highest level of greenness (0.76) compared to the reported methods [9,10,11], which had greenness levels of 0.66, 0.57, and 0.52, respectively. Among the HPLC-UV methods, the LC-MS/MS method [11] had the lowest greenness level due to its higher energy consumption. The superior greenness level of the proposed MW-SPMs in comparison to the reported methods could be primarily attributed to the sample volumes and, consequently, the waste generated. In the proposed method, the waste produced per sample was 200 µL, whereas the reported liquid chromatographic methods had waste volumes ranging from 4 mL to 8 mL per sample. Additionally, the reported HPLC-UV and LC-MS/MS methods employed larger volumes of solvents, such as methanol, acetonitrile, formic acid, and different buffers in the mobile phase, which had a negative impact on their greenness levels. In contrast, the proposed MW-SPMs utilized smaller volumes of samples or reagents (totaling 200 µL per sample).

### 2.9. Throughputs of the MW-SPMs

Microwell methods have garnered considerable attention in pharmaceutical analysis due to their capacity to analyze and manipulate numerous individual entities simultaneously and with high throughput. These methods involve the utilization of arrays consisting of microscale wells or compartments, where each well can accommodate a single entity, such as a targeted analyte molecule [13,14,15]. The throughputs of the proposed MW-SPMs were evaluated based on the use of 96-well plates and a reaction time of 10 min. Under these conditions, it was feasible for an analyst to comfortably process at least five plates as a batch. Consequently, 2400 samples were processed per hour (5 plates × 96 wells × 5 rounds/h). This high throughput could be further enhanced through various approaches; these approaches encompass the utilization of plates with a greater number of wells (384, 1536, or 3456 wells) and the automation of the process using robots.

## 3. Experimental Procedure

### 3.1. Apparatus

The double-beam ultraviolet–visible spectrophotometer (model V-530) was a product of JASCO Co. Ltd. (Kyoto, Japan). It was used for scanning the absorption spectra of CER and its reaction mixtures. The absorbance microplate reader (model ELx808) used for these experiments was a product of Bio-Tek Instruments Inc. (Winooski, VT, USA). The instrument was controlled via KC junior software (version 2.0) provided with the reader. The reader was featured with a built-in temperature control system, which enables the heating and temperature maintenance of solutions in microwell plates at the desired temperature up to 50 °C. A top-reading mode was employed for absorbance readings using the reader. Additionally, the reader can perform a 1 min shaking action to thoroughly mix the reagents with the analyte solutions. After shaking, a brief settling time of 300 milliseconds was allowed before measuring the absorbances.

### 3.2. Chemicals and Materials

The standard CER material was purchased from LC Laboratories (Woburn, MA, USA); its purity was ≥99%. Zykadia^®^ capsules (Novartis Pharmaceutical Corporation (Basel, Switzerland) were kindly donated by Saudi FDA (Riyadh, Saudi Arabia), labeled to contain 150 mg CER per capsule. ACD, CHL, BRL, and DCNQ were purchased from Sigma-Aldrich Chemicals Co. (St. Louis, MO, USA). The concentration of the ACD solution was 2% (*v*/*v*, in methanol). The concentrations of the CHL, BRL, and DCNQ solutions were 5% (*w*/*v*, in methanol). All solutions were prepared fresh in a working day. Transparent polystyrene Corning^®^ 96-well plates were obtained from Merck & Co., Inc. (Rahway, NJ, USA). Adjustable single and 8-channel pipettes, branded Finnpipette^®^, were obtained from Thermo Fisher Scientific Inc. (Waltham, MA, USA). To dispense solutions using the 8-channel pipettes, reagent reservoirs with cover lids were purchased from Merck KGaA (Darmstadt, Germany), and were of the BRAND^®^ PP variety. All solvents used were of spectroscopic grade and were supplied by Merck (Darmstadt, Germany). All chemicals used in this study were of analytical grade.

### 3.3. Preparation of Standard and Capsule Solutions

For preparation of a standard CER solution, a weighed quantity (20 mg) of CER powder was dissolved in 10 mL of methanol. This stock solution (2 mg/mL) was diluted with methanol to yield working solutions in concentration ranges of 5–150 µg/mL.

For the preparation of the capsule solution, the contents of 10 Zykadia^®^ capsules were collected, and an accurate weight of the contents equivalent to 50 mg of CER was transferred to a 25 mL calibrated flask. Following this step, 20 mL of methanol was added, and the contents were shaken well to ensure the complete dissolution of CER. The flask’s content was filtered via passing through 0.4 μm membrane filter. The filtrate was diluted with methanol for analysis via the MW-SPMs. 

### 3.4. Optimization of Experimental Conditions

#### 3.4.1. Concentration of ACD

The optimal concentration of ACD was determined by dispensing 50 µL of varying concentrations (0.05–8%, *v*/*v* in methanol) into each well of the 96-well plate that already contained 100 µL of the standard CER solution (25 μg/mL). In each well, CHL, BRL, or DCNQ reagent solution (5%, *w*/*v*) was added, and the reaction proceeded at room temperature (25 ± 2 °C) for 10 min. The absorbance of the colored mixed solution in each well was measured using the reader at 665 nm, 655 nm, and 580 nm for the reaction mixtures involving CHL, BRL, and DCNQ, respectively. To prepare the blank wells, the same procedure was followed, with the exception that 100 μL of methanol was dispensed into the wells instead of the standard CER solution.

#### 3.4.2. Concentrations of CHL, BRL, and DCNQ

The optimal concentrations of the CHL, BRL, and DCNQ solutions were determined by adding 50 µL of varying concentrations (0.5–12%, *w*/*v*) of each reagent solution into each well of the plate containing 100 µL of CER solution (25 μg/mL) and 50 μL of ACD solution (2%, *v*/*v*). The plate was further manipulated using the same procedure described in optimizing the concentration of ACD.

#### 3.4.3. Reaction Time and Temperature

The optimal amount of time required for completing the reaction was tested by checking the color development at room temperature (25 ± 2 °C) and at 50 °C, using a kinetic and temperature control feature of the reader. The concentrations of CER, ACD, and haloquinone reagents (CHL, BRL, and DCNQ) were 25 μg/mL, 2% (*v*/*v*), and 5% (*w*/*v*), respectively.

#### 3.4.4. Solvent

The best solvent for conducting the reactions was selected by executing the reactions (using the above-mentioned CER and reagent concentrations) at room temperature (25 ± 2 °C) in different solvents. These solvents were methanol, ethanol, propanol, butanol, acetonitrile, acetone, chloroform, dichloroethane, and dioxane.

### 3.5. Procedures Underlying the MW-SPMs and Construction of Calibration Curves

Standard CER solution (100 µL) was transferred into each well of the plate. To each well, 50 μL of ACD solution (2%, *v*/*v*), followed by 50 μL of haloquinone reagent (CHL, BRL, or DCNQ) solution (5%, *w*/*v*) were added. The reaction proceeded at room temperature (25 ± 2 °C) for 10 min. The reader was then used to measure the absorbance in each well at 665 nm, 655 nm, and 580 nm for reactions with CHL, BRL, and DCNQ, respectively. The blank wells were treated in a similar way, with the exception that 100 μL of methanol was dispensed in each well instead of the standard CER solution. The absorbance readings of the blank wells were subtracted from those of the test solutions. The resulting difference in absorbances was plotted against the corresponding concentrations of CER to generate calibration curves. Regression analysis of the data was performed, and linear fitting equations were generated along with their parameters (intercepts, slopes, and determination coefficients). Capsule sample solutions were analyzed using the same procedures, and the linear fitting equations were utilized to determine the contents of CER in the capsule sample solutions.

### 3.6. Content Uniformity Testing of Zykadia^®^ Capsules

The procedure outlined in the United States Pharmacopeia (USP) [31] was employed to conduct content uniformity testing on Zykadia^®^ capsules. The analysis involved using the proposed MW-SPMs to analyze 10 capsules individually, measuring the content of CER in each individual capsule, as well as determining the average content across the 10 capsules. Subsequently, the acceptance value was calculated and compared to the maximum allowable value specified for content uniformity testing of 10 units of solid dosage form [31].

## 4. Conclusions

This study focused on the development and validation of three MW-SPMs for quantifying CER in capsule formulations. These methods involved the formation of colored N-vinylamino-substituted haloquinone derivatives of CER upon its reactions with CHL, BRL, and DCNQ, in the presence of ACD. Transparent 96-well plates were used to conduct the reactions, and the absorbances of the resulting-colored solutions were measured using an absorbance microplate reader. These methods were novel and specifically designed for CER, offering simple procedures that adhered to the requirements of GAC practices in quality control laboratories within the pharmaceutical industry in terms of their eco-friendliness and health safety. These three MW-SPMs were successfully applied to determine the CER content in capsules and perform drug uniformity testing. Additionally, they demonstrated high analytical throughput, allowing an analyst to comfortably analyze approximately 2400 samples per hour. Overall, this work expands the possibilities for the efficient utilization of microwell assays aided using plate readers in drug quantitation. It highlights the practical applications of these methods in the pharmaceutical industry, demonstrating their convenience and efficacy.

## Figures and Tables

**Table 1:** Optimization of the experimental conditions of the MW-SPMs for the determination of CER via its reactions with ACD and haloquinone reagents: CHL, BRL, and DCNQ.

Condition	Studied Range	Optimum Value
ACD conc. (%, *v*/*v*)	0.1–8	2
CHL, BRL, and DCNQ conc. (%, *w*/*v*)	0.5–12	5
Solvent	Different ^a^	Methanol
Reaction time (min)	0–30	10
Temperature (°C)	25 and 50	25
λ_max_ (nm)	400–900	665, 655, and 580 ^b^

^a^ The solvents used were methanol, ethanol, propanol, butanol, acetonitrile, acetone, chloroform, dichloromethane, and dioxane. ^b^ The reported λ_max_ values were for reactions with CHL, BRL, and DCNQ.

**Table 2:** Regression and statistical parameters for the determination of CER using the proposed MW-SPMs via reactions with ACD and haloquinone reagents: CHL, BRL, and DCNQ.

Parameter	Value		
	CHL	BRL	DCNQ
Linear range (µg/mL)	5–100	5–120	5–150
Intercept	0.0168	0.0238	0.0005
Slope	0.0208	0.0163	0.0129
Determination coefficient (r^2^)	0.9995	0.9992	0.9991
Limit of detection (µg/mL)	1.6	1.8	2.3
Limit of quantitation (µg/mL)	5.3	5.9	7.6

**Table 3:** Accuracy and precision of the proposed MW-SPMs for the determination of CER via its reactions with ACD and haloquinone reagents: CHL, BRL, and DCNQ.

CER Conc. (µg/mL)		Recovery (%) ^a^		
		CHL	BRL	DCNQ
Intra-day				
20		99.6	101.2	100.2
40		100.8	98.9	99.8
100		101.4	99.7	101.7
	Average	100.6	99.9	100.6
	RSD (%)	0.9	1.2	1.0
Inter-day				
20		101.2	99.4	102.2
40		102.3	98.8	101.4
100		99.5	101.4	98.7
	Average	101.0	99.9	100.8
	RSD (%)	1.4	1.4	1.8

^a^ Averages of three and six determinations for intra- and inter-day analysis, respectively.

**Table 4:** Robustness and ruggedness of the proposed MW-SPMs for the determination of CER via its reactions with ACD and haloquinone reagents: CHL, BRL, and DCNQ.

Parameter	Change Range	Recovery (% ± RSD) ^a^		
		CHL	BRL	DCNQ
Robustness				
Recommended conditions ^b^	No change	99.5 ± 1.5	100.3 ± 0.9	99.3 ± 0.6
ACD conc. (%, *v*/*v*)	1.5–2.5	100.6 ± 1.4	99.2 ± 1.7	98.8 ± 1.5
Haloquinone reagent conc (%, *w*/*v*)	4–6	99.1 ± 1.6	100.5 ± 0.7	100.5 ± 0.9
Reaction time (min)	8–15	97.4 ± 1.5	102.9 ± 1.5	101.3 ± 1.6
Ruggedness				
Analyst-to-analyst	3 Analysts	100.8 ± 1.8	101.4 ± 0.7	100.3 ± 1.7
Day-to-day	3 Days	100.5 ± 1.1	99.8 ± 0.8	101.5 ± 1.5

^a^ Values are means of 3 determinations. ^b^ The recommended conditions were outlined in Table 1.

**Table 5:** Applications of the MW-SPMs for the determination of CER in Zykadia^®^ capsules via its reactions with ACD and haloquinone reagents: CHL, BRL, and DCNQ.

Nominated CER conc. (µg/mL)		Label Claim (% ± RSD) ^a^		
		CHL	BRL	DCNQ
20		100.2 ± 1.4	101.5 ± 1.2	99.8 ± 0.9
40		99.8 ± 1.2	98.9 ± 1.1	100.2 ± 1.4
100		100.4 ± 1.5	100.6 ± 0.9	101.6 ± 1.5
	Mean	100.1 ± 0.3	100.3 ± 1.3	100.5 ± 0.9

^a^ Average of three determinations.

**Table 6:** Content uniformity testing of Zykadia^®^ capsules using the proposed MW-SPMs for the determination of CER via its reactions with ACD and haloquinone reagents: CHL, BRL, and DCNQ.

Capsule Number	Label Claim (%)		
	CHL	BRL	DCNQ
1	101.5	100.5	99.6
2	98.8	103.2	99.5
3	98.7	102.6	102.8
4	100.2	98.5	101.7
5	103.5	97.6	103.2
6	102.4	100.4	102.8
7	99.7	98.7	101.6
8	97.5	100.5	99.8
9	100.8	96.8	96.4
10	102.4	99.8	97.5
Mean	99.6	99.9	100.5
SD	1.9	2.0	2.3
Acceptance value	4.6	4.9	5.6
Maximum allowed value	15	15	15

**Table 7:** The analytical eco-scale approach for assessing the greenness of the proposed MW-SPMs for the determination of CER.

Eco-Scale Score Parameters	Penalty Points (PPs)
Amount of solvent/reagent	
Solvent: <1 mL (mL (g) per sample)	1
Reagent: <1 mL (mL (g) per sample)	1
	∑ = 2
Hazard of solvent/reagent	
Solvent: methanol	3
Reagent: CHL, BRL, and DDQ	3
	∑ = 6
Instrument: Energy used (kWh per sample)	
Microplate reader	0
	∑ = 0
Occupational hazard	
Analytical process hermetic	0
Emission of vapors and gases to the air	0
	∑ = 0
Waste	
Production (<1 mL (g) per sample)	1
Treatment (no treatment involved)	3
	∑ = 4
Total PPs	12
Eco-scale score	88

## Data Availability

All data are available from the corresponding author (idarwish@ksu.edu.sa).
